# Stroke Management: An Emerging Role of Nanotechnology

**DOI:** 10.3390/mi8090262

**Published:** 2017-08-28

**Authors:** Deepaneeta Sarmah, Jackson Saraf, Harpreet Kaur, Kanta Pravalika, Rakesh Kumar Tekade, Anupom Borah, Kiran Kalia, Kunjan R. Dave, Pallab Bhattacharya

**Affiliations:** 1 Department of Pharmacology and Toxicology, National Institute of Pharmaceutical Education and Research (NIPER), Ahmedabad, Gandhinagar, Gujarat 382355, India; deepa92sarmah@gmail.com (D.S.); jumbo1824@gmail.com (J.S.); hksaini1993@gmail.com (H.K.); Kantapravallika@gmail.com (K.P.); kirankalia@gmail.com (K.K.); 2 Department of Pharmaceutics, National Institute of Pharmaceutical Education and Research (NIPER), Ahmedabad, Gandhinagar, Gujarat 382355, India; rakeshtekade@gmail.com; 3 Cellular and Molecular Neurobiology Laboratory, Department of Life Science and Bioinformatics, Assam University, Assam 788011, India; anupomborah@gmail.com; 4 Department of Neurology, University of Miami Miller School of Medicine, Miami, FL 33136, USA; KDave@med.miami.edu; 5 Department of Neurosurgery, Boston Children’s Hospital, Harvard Medical School, Boston, MA 02115, USA

**Keywords:** stroke, nanotechnology, diagnosis, therapy, neuroprotection

## Abstract

Stroke is among the leading causes of mortality and morbidity worldwide. Stroke incidences and associated mortality are expected to rise to 23 million and 7.8 million, respectively, by 2030. Further, the aging population, imbalanced lifestyles, and environmental factors continue to shift the rate of stroke incidence, particularly in developing countries. There is an urgent need to develop new therapeutic approaches for treating stroke. Nanotechnology is a growing field, offering an encouraging future prospect for medical research in the management of strokes. The world market for nanotechnology derived products is expected to rise manyfold in the coming decades. Different types of nanomaterials such as perfluorocarbon nanoparticles, iron oxide nanoparticles, gold nanoparticles, polymeric nanoparticles, quantum dots, nanospheres, etc. have been developed for the diagnosis as well as therapy of strokes. Today, nanotechnology has also been integrated with stem cell therapy for treating stroke. However several obstacles remain to be overcome when using such nanomaterials for treating stroke and other neurological diseases.

## 1. Introduction

As a leading cause of mortality worldwide, the burden of stroke threatens to amass drastically in the coming years. Extrapolating from the neuroepidemiology of 2005, cases of stroke are predicted to rise to 23 million and 7.8 million related deaths in 2030 [1]. However, these statistics remain tentative as the task of orchestrating a major global survey of stroke incidence is exhaustive and even more expensive. Still, an attempt was made by the 2013 Global Burden of Disease (GBD) study, revealing stroke to be the second leading cause of death behind only cardiovascular diseases [2].

The aging population, lifestyle, and environmental factors continue to shift the rate of stroke incidence further, particularly in developing countries. The elderly population is expected to outnumber the younger population by 2050 [3]. This raises concern as studies have already shown the drastic rise in stroke risk beyond the age of 60; changes in the brain after 60 years of age are nearly double that between 20 and 60 years of age [4,5]. However, what once was considered mainly a disease of the elderly has now been evidenced to occur in two-thirds of the population below 70 years of age [6].

Risk factors such as hypertension, dyslipidemia, diabetes, smoking, poor diet, and lack of physical activity constitute up to 80% of stroke cases [7]. Despite stroke being a non-communicable and preventable disease, it has become highly prevalent throughout the world [8]. Thromboembolism remains the major contributor to the underlying pathophysiology. The occlusion in the cerebrovasculature hinders the blood flow supplying the brain. This renders an ischemic condition, which starves the cerebral neuronal environment of adenosine triphosphate (ATP) and disrupts energy-requiring cellular processes that are essential for cell survival. This further culminates in infarcts in different brain regions and neuronal degeneration that elicits cognitive and motor deficits [9,10]. Other etiologies causing ischemic stroke are systemic inflammatory processes such as vasculitis, rheumatoid arthritis, and cerebral vasospasms [11].

Haemorrhagic strokes are more common in arteriosclerosed vasculature, accelerated by lipohyalinosis [12]. Aneurysms and the rupturing of intracranial blood vessels present a dangerous situation as intraparenchymal blood triggers pathological factors such as blood cytotoxicity, oxidative stress, and inflammation, ultimately damaging the blood-brain barrier (BBB) and potentially causing brain edema in which massive neuronal degradation ensues. In such situations, timely removal of the damaged neuronal tissue dictates patient survival [13,14,15,16].

The complexity of the molecular mechanisms as well as their physiological ramifications requires therapy that will focus on controlling and mitigating the cascade of apoptotic events that contribute to neuronal cell death. Despite the progress in stroke diagnosis and treatment [17], a quest for superior alternatives that help detect early changes in the cerebrovasculature and also form the foundation of not only salvaging but protecting the neuronal environment from ischemic insult have now directed stroke research toward the promising field of nanomedicine [18].

## 2. Advent of Nanotechnology

Nanotechnology in the field of medical research is offering an encouraging perspective in the therapy of various central nervous system disorders [19]. Nanotechnology is considered to be the generation and application materials or devices at the nanoscale level. Nanomaterials are considered to entail those materials that have single units with at least one dimension between 1 and 100 nm [20]. The concept and the origin of nanotechnology are attributed to Richard P. Feynman. The term ‘nano’ is derived from the Greek word meaning ‘dwarf’. A nanometer may be defined as one-billionth of a meter or the length of 10 hydrogen atoms placed side by side or 1/80,000th of the thickness of human hair. In medicine, nanoparticles (NPs) range in size from 5 to 250 nm [19]. Advocators promise to maneuver nanotechnology in such a manner as to spark a wave of novel and revolutionary products from machines to medicine [21]. NPs are a result of numerous physical or chemical processes, and these possess specific properties [22].

The world market for nanotechnology derived products is expected to increase by a large amount in the coming decades. The Indian government is also investigating and committing to nanotechnology applications in medicine, engineering, biotechnology, space research, etc. The Indian and Australian governments together contributed nearly 20 million dollars to start an Australia-India Science Research Funding Programme (AISRF). The global market value of the nanomedicine industry was estimated to be $63.8 billion in 2010 and increased to $72.8 billion in 2011, as published in a report by BCC Research [19,23].

Strokes have a greater disability impact than other disorders, according to the reports published by The American Stroke Association (ASA) and The Stroke Association UK. As reported by Saver, nearly 1.2 billion neurons, 8.3 trillion synapses, and 4470 miles or 7140 km of myelinated fibers are lost during a stroke episode. Following an ischemic attack, where there is delay in receiving treatment, it is estimated that the number of neurons lost is equivalent to the number of neurons lost in 36 years of normal ageing [18,24]. This fact has inspired numerous researchers to look out for alternative therapies, as the currently employed methods are limited in terms of earlier detection and treatments. For this reason, cerebrovascular and cardiovascular nanotechnology shows promise in managing the current difficulties [18,19].

## 3. Nanotechnology for the Diagnosis of Stroke

**Figure 1:**
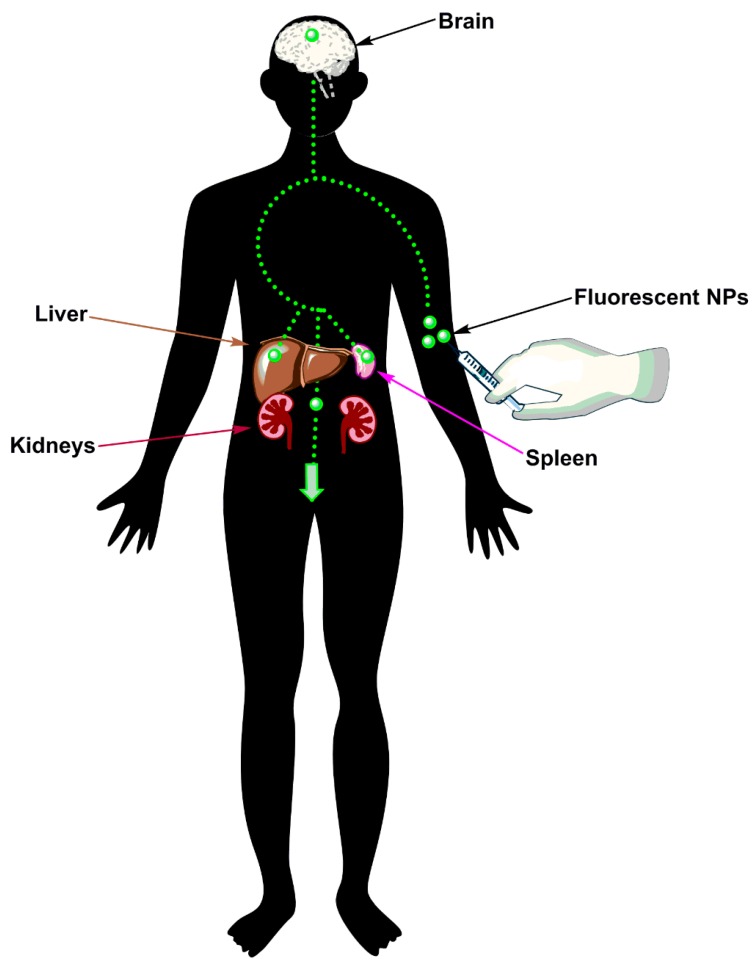
Nanoparticles (NPs) used for diagnosis. Some NPs such as quantum dots can be employed for bio-imaging due to their inherent fluorescent nature and their use as contrasting agents. This helps to track them in vivo and to understand their pharmacokinetics. Although the kidneys serve as a primary route of clearance, the liver and spleen also serve to clear NPs from the body.

Recent research has exhibited the promising application of NPs to stroke therapy [25]. Magnetic resonance imaging (MRI), computed tomography (CT), positron emission tomography (PET), and ultrasound are employed for the diagnosis of stroke. Although highly beneficial, some restrictions of these techniques exist. Hence this has led many research groups to unfold more advanced imaging techniques with the help of nanotechnology (Figure 1) [26].

NPs are used as an efficient drug delivery system, the characteristics of which include having a diameter less than 100 nm, being non-toxic and biocompatible, having a biodegradable nature, being stable in blood, being capable of BBB-targeting, being non-inflammatory, and having a prolonged circulation time [18]. Biomarkers are regularly being used for improving stroke diagnosis. S100 calcium binding protein B (S100B), glial fibrillary acidic protein (GFAP), and vascular cell adhesion molecule (VCAM) are some of the biomarkers that have been well studied in strokes. These biomarkers can be incorporated with NPs for quick detection by integrating other techniques such as CT and MRI [27].

**Figure 2:**
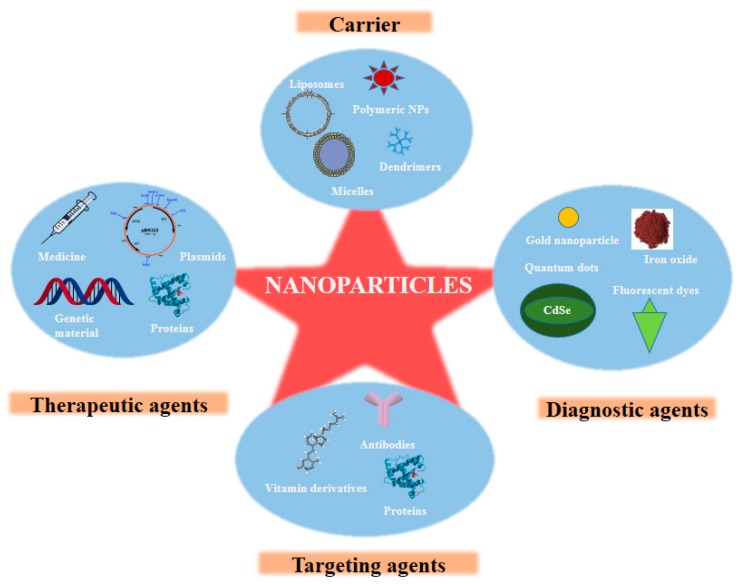
Nanoparticles in the management of stroke.

Different types of NPs, which have been utilized for diagnosis, are perfluorocarbon NPs, iron oxide NPs, gold NPs, polymeric NPs, quantum dots, nanospheres, etc. (Figure 2) [28].

### 3.1. Perfluorocarbon Nanoparticles (PFC-NPs)

Perfluorocarbon NPs (PFC-NPs) are non-metabolizable, non-toxic, and inert. PFC-NPs can target epitopes and at the same time, be integrated on the NP surface and can carry a high paramagnetic payload of 60,000 to 90,000 gadolinium ions per particle. Therefore, it is a very sensitive detector to target epitopes with MRI [29]. α_v_β_3_-integrin-targeted PFC-NPs were used to detect the early manifestations, along with MRI [30]. The neurovascular density is proportional to the magnitude of the MRI signal, and PFC-NPs can enhance this magnitude. Ligand-directed PFC-NPs show promising results for the early and accurate detection of therapeutic responses in patients with stroke [31].

### 3.2. Iron Oxide NPs

Superparamagnetic iron oxide NPs (SPIOs) have been successfully utilized as MRI contrast agents. There are some SPIOs available in the market such as ferumoxide coated with dextran (SPIO), ferumoxytol coated with poly-glucose sorbitol carboxymethyl ether (USPIO), and VSOP-C184 coated with citrate (VSPIO) [32]. They have the advantage of being non-toxic, having long circulating blood half-life, the ability to penetrate the BBB, a low side effect profile, possible pathological identification, anatomical targeting, and clearance by phagocytic cells [33]. However, a limitation of not being able to differentiate the resident iron signals of the brain from the USPIO signals exists [26].

### 3.3. Gold NPs (GNPs)

Intravascular photoacoustic (IVPA) imaging is an invasive, catheter-based imaging technique, which images the optical absorption of atherosclerotic tissue using ultrasound. GNPs are used as contrast agents for IVPA to detect macrophages in atherosclerotic plaques [34]. However, delivering large-sized GNPs may become a risk factor. For instance, for delivering 50 nm gold NPs coated with thiolated polyethyleneglycol (PEG), a MRI-guided focused ultrasound technique can be utilized [13]. PEG-coated GNPs can cross the BBB, have improved solubility and stability, and show better uptake kinetic profile [35].

### 3.4. Polymeric NPs

Polymeric NPs are made up of biodegradable polymers [poly-d, l-Lactide-co-glycolide (PLGA)], which can entrap agents that are hydrophilic. They can deliver drugs by getting internalized into the cells via endocytosis. These are also used to coat metallic stents. PLGA NPs have been encapsulated with fluorescein isothiocyanate (FITC), which is a hydrophilic dye, into balloon-expandable stents. Efficient delivery of the FITC-encapsulated NPs was observed when a coronary smooth muscle was incubated with it [35]. Lu et al., have recently designed cationic polymeric micelles, which were loaded with different dyes to label neural stem cells (NSCs). Their study showed that such micelles showed high efficacy, safety, and reliability in in vivo tracking of stem cells in stroke therapy using MRI [36]. Similarly two other research groups developed polymersomes, which constitute a type of polymeric NP, for the imaging of therapeutic stem cells using MRI in case of stroke [37,38].

Another important component of NPs is nanospheres. A drug can be uniformly dispersed into a nanosphere matrix [39]. Nanospheres, due to their large surface area, exhibit target-specific drug delivery. They also demonstrated neuroprotective properties [40]. Caspase-3 inhibition improves cell survival after ischemia. Nanospheres loaded with Z-DEVD-FMK showed a considerable decrease in the neuro-deficits and activity of caspase-3, reduced the infarct volume, and may help in the prevention of stroke [41].

### 3.5. Quantum Dots

Quantum dots are optical contrast agents, employed as drug delivery and bioimaging agents for labeling molecules, cells, and tissues [42]. They consist of a semiconductor core and encompass elements from groups II to VI of the periodic table, of which cadmium selenide (CdSe) is the most commonly used. Quantum dots offer stable fluorescent tags, enhanced optical properties, and a composition that can be adjusted [43]. However, toxicity is associated when these are directly injected into the vasculature, and systemic toxicity is shown when CdSe quantum dots are illuminated with ultraviolet light. Quantum dots also show toxicity towards cultured cells [44]. 

## 4. Stroke Therapy Using Nanotechnology

The need of the hour is to develop new therapeutic approaches for the treatment of stroke, and the field of nanotechnology is proving to be a promising approach for stroke treatment. Adenosine has shown to be beneficial in various neurological conditions. When converted to nano-assemblies by conjugating adenosine with liquid squalene, improvement in neuro deficits has been shown in an animal model of ischemia [45]. Lee et al. pretreated rats with amine-modified single walled nanotubes and found that such rats have decreased levels of neuronal apoptosis markers. Also reduced glial and inflammatory responses post-stroke indicate that such nanotubes limit cell death and inflammation [46]. The group also noted that N-cadherin levels, an essential protein for cell adhesion and survival, remained high in animals treated with nanotubes. It has been reported that neurons showed a 200% increase in neurite length when they were grown on multi-walled carbon nanotubes (MWCNT), while there was up to a 300% increase on coated MWCNT compared to uncoated nanotubes [47]. MWCNTs are biocompatible and effective transporters for small interfering RNA (siRNA) that can silence apoptotic genes, which provides a potential therapeutic use of CNT in stroke [18].

Nanotechnology has been integrated with cell therapy, which has been extensively employed for the treatment of stroke. Iron-labeled neural progenitor cells (NPCs) showed migration towards the ischemic region and promoted angiogenesis when transplanted into the cisterna magna of rats with middle cerebral artery occlusion (MCAO), similarly such cells also showed migration towards the ischemic boundary, where they enhanced angiogenesis [48,49]. This migration of transplanted cells has been visualised by MRI [23]. Nanofibres produced by the electrospinning technique have been shown to support stem cell proliferation and growth, neurite outgrowth, and glial migration [50,51,52,53]. Tysseling et al. [54] reported that self-assembling nanofibres significantly stimulated behavioral improvement in animal stroke models.

The antioxidant properties of cerium oxide (CeO) NPs promoted the survival of cells and significantly reduced free radical production [18]. In an animal model of stroke, CeO NPs reduced cell death significantly [55]. They were also found to reduce the levels of ischemia-generated 3-nitrotyrosine, induced by a peroxynitrite radical [55]. Recently, platinum (Pt) NPs have been employed as free radicals and reactive oxygen species scavengers in vitro and in vivo in mice, thereby ameliorating the oxidative stress generated in ischemic conditions [56]. In the mouse model of MCAo, Pt NPs improved motor functioning and decreased the volume of infarct in the cortex [57]. These NPs have also been shown to successfully inhibit MMP-9 activation, thereby promoting neurological improvements [57,58]. Tissue plasminogen activator (tPA) is known to induce pathological and clinical damage, which was reduced by the use of Pt NPs, owing to the virtue of their catalase and superoxide dismutase-mimicking activity [59].

Xenon-encapsulated liposomes have been therapeutically delivered to the brain for stroke therapy using ultrasound guidance as xenon has been demonstrated to have neuroprotective properties [60]. Nanospheres and polymeric NPs are also increasingly being used as agents for ischemic stroke therapy [35,61].

## 5. Challenges

**Figure 3:**
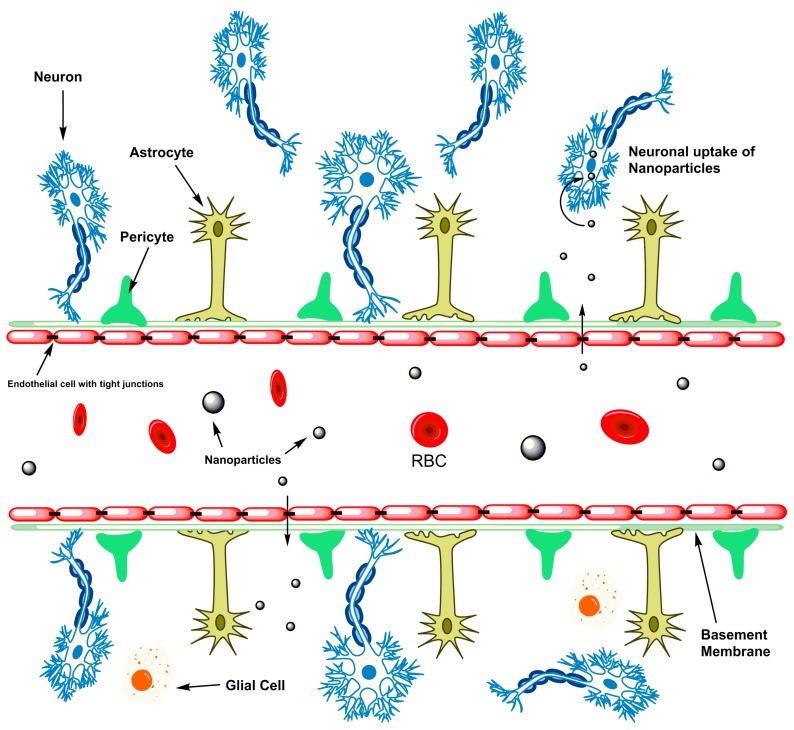
NPs are able to cross the restrictive blood brain barrier to be taken up by neurons and prevent further neurodegeneration.

Nanotechnology research has exponentially grown within the last few decades, and the focus on brain disorders and stroke has increased in parallel. Theranostic development has led to a significant amount of understanding of some of the complex etiologies involved as well as increasing the chances of early diagnosis and therapeutic potential with the help of nanomedicine [62]. Several obstacles do, however, remain when it comes to treating stroke and other neurological diseases. The greatest hurdle by far is the BBB, a restrictive and essential component of protecting the brain from any harmful substances circulating in the blood and of blocking the entry of small molecules and mostly all macromolecules (Figure 3) [63]. Although well documented and studied, the entire composition and physiology of the BBB are still not completely elucidated. Part of this may be due to the complexity of enzyme transporters and the tight and adherent junctions spaced throughout the brain’s endothelial lining [64,65].

The other hurdle is tied to the leaky vasculature and BBB observed in stroke. As the BBB is unable to function optimally, there is greater chance that nanomaterials aimed to reach the brain end up reverting to systemic circulation. Although a leaky BBB initially facilitates an easier NP delivery to the brain, it may also cause a regurgitation of the administered dose back into the systemic circulation to precipitate side effects. This raises a challenge on how to exploit the leaky BBB while simultaneously mitigating the backflow. Furthermore, some NPs may even exacerbate BBB leakage [66]. A recent study by Sharma et al. displayed the breakdown of BBB membrane integrity and induced brain edema when the BBB was treated with different nanomaterials [67]. The leaking of NPs back into systemic circulation also implicates further complications; nanomaterials have shown a tendency to cause haemolysis, which can spell disaster by causing anaemia or reticulocytosis, both of which can be life-threatening [68]. To perpetuate this problem further, the lysed haemoglobin and cell debris may attach to circulating nanomaterials and trigger an immune cascade, which has been previously shown to be a cause of non-cerebrovascular occlusive stroke [69].

**Figure 4:**
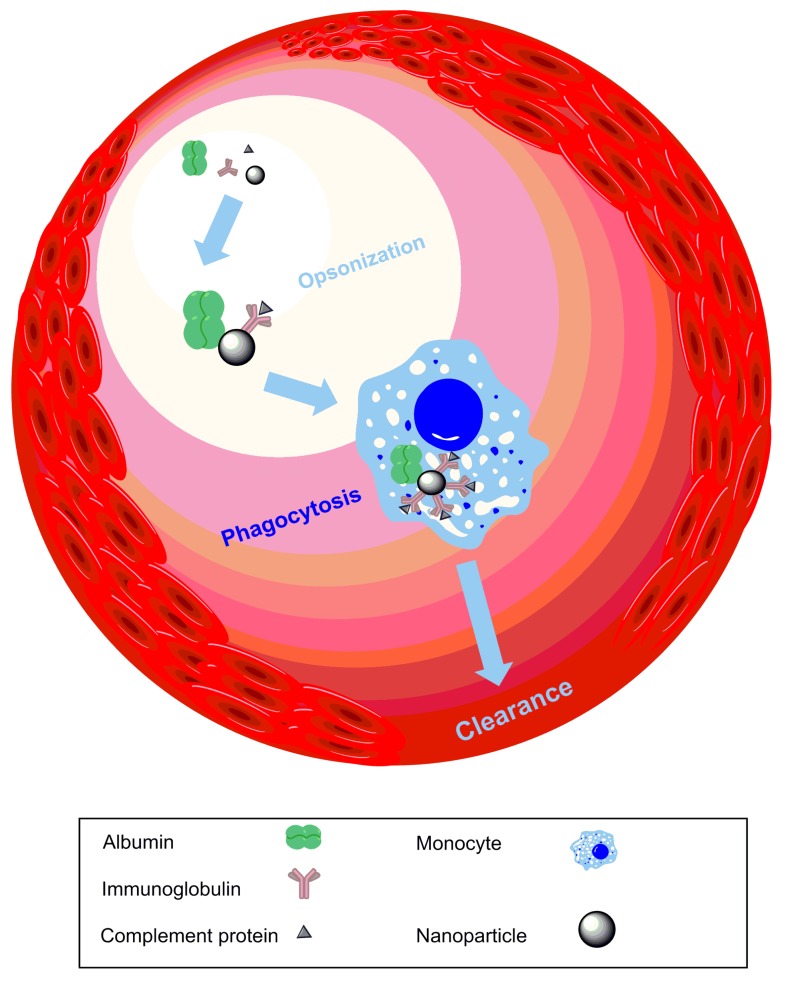
Challenges with NPs: prolonged circulation exposes NPs to opsonisation by albumin, phagocytosis by monocytes, and clearance from the body, thereby reducing their bioavailability and overall therapeutic effect.

Another issue when designing nanomedicine to treat stroke is that the circulation time must be prolonged to increase the fraction of nanomaterial reaching the brain. In doing so, there is greater exposure to circulating coagulation factors, which can increase platelet aggregation and thrombus formation. This would further exacerbate the condition by creating more blood vessel occlusions [41], and, in severe conditions, dissemination due to intravascular coagulation may occur [70]. Increased circulation time can also reduce the bioavailability of nanomedicine due to an acquired antigenic property after opsonization by plasma proteins and thus enhanced clearance by phagocytes and the reticuloendothelial system (Figure 4). 

A final major concern is the lack of protocols to assess the cytotoxicity of nanomaterials [71]. Various studies have cited inflammation and free oxygen radical generation in response to different nanomaterials [72]. Therefore, specific factors like cell type, the dosage administered, the effect of the biological environment on nanomaterial physicochemical properties, and the most relevant cytotoxic assay must be appropriately chosen and taken into consideration in order to study nanomaterial cytotoxicity. A failure to accurately assess the toxic profile of nanomaterials on a greater level continues to pose toxicity concerns when moving from bench to bedside [73].

## 6. Conclusions

**Figure 5:**
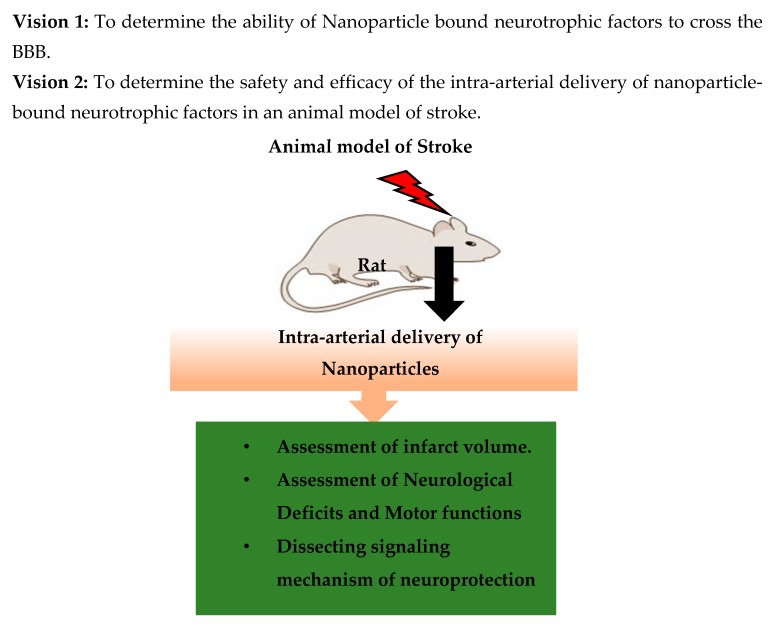
The vision for our future research.

The increasing threat of global stroke morbidity and mortality, the shortcomings and risks displayed by the incumbent therapies, and the desire for more efficient and safer alternative therapies for treating stroke have steered research towards nanotechnology. The complex nature of stroke and the difficulties in early detection and poor prognosis have stirred an urgency to develop better theranostic alternatives. Various nanomaterials offer greater options for early diagnosis and more lucid clarification of the pathophysiology. A deeper comprehension of early stress signals in the cerebrovasculature may help to prevent the impending cerebral ischemia as well as reduce the degree of damage a patient may suffer. With greater drug delivery efficacy, more efficient drug delivery may be accompanied by safer dosing and, subsequently, fewer side effects known to hinder the current drug therapies used for stroke. Of course, nanotechnology does not come without its caveats. Thus, nanomedicine targeting stroke treatment must be meticulously designed to achieve the safest and most efficacious therapeutic regimen. It is with this vision that nanotechnology pushes forward to develop more promising stroke therapies, and researchers may be able to use it as a tool to deliver different neuroprotectants to the brain by combating the BBB (Figure 5).
